# Needs of family members caring for stroke survivors in china: A deductive qualitative content analysis study by using the caregiver task inventory-25

**DOI:** 10.1186/s12877-022-02774-5

**Published:** 2022-02-03

**Authors:** Qi Lu, Jan Mårtensson, Yue Zhao, Linda Johansson

**Affiliations:** 1 School of Health and Welfare, Jönköping University, Jönköping, Sweden; 2 Present Address: School of Nursing, Tianjin Medical University, Tianjin, China

**Keywords:** Deductive content qualitative analysis, Family caregiver, Needs, Stroke

## Abstract

**Background:**

In China, family caregivers are usually the main carers of relatives after stroke due to traditional Chinese culture and the limited development of the primary healthcare system. This responsibility often results in burdens and negative health outcomes. However, family caregivers seldom receive support. To improve informal care, as well as the health and well-being of family caregivers, it is important to identify their needs.

**Objective:**

This study aimed to deductively explore the needs of family members caring for stroke survivors in China.

**Methods:**

Twenty-six semi-structured interviews were performed with family caregivers of stroke survivors who were selected from one city and three communities by purposive sampling. A deductive qualitative content analysis method was performed by using the Caregiver Task Inventory-25 (CTI-25), an instrument measuring the needs of family caregivers, as a framework.

**Results:**

All subscales, as well as all belonging items in the CTI-25, were identified in the present study, meaning that the family caregivers had needs related to *learning to cope with new role, providing care according to care-receiver’s needs, managing own emotional needs, appraising supportive resources, and balancing caregiving needs and one’s own needs*. Moreover, needs related to financial support, both direct and indirect, were identified but not part of the CTI-25.

**Conclusion:**

These findings identified that family caregivers of stroke survivors in China had various needs, which is important knowledge when assessing needs and improving health care for family caregivers. Cultural adjustments and modifications should be made if CTI-25 is used in mainland China. This study also indicated a comprehensive and holistic perspective (individual, community, and social level) when identifying, assessing needs or implementing interventions to support family caregivers.

## Introduction

Stroke carries a high risk of death and permanent disability, especially in developing countries. From 1990 to 2016, the global lifetime risk of stroke increased from 22.8% to 24.9%, and China was found to have the highest risk with 39.3% [1]. It is estimated that the age-standardized prevalence of stroke in China accounts for 1.1% [2], which means that stroke is the leading cause of years of life lost (YLLs) and disability-adjusted life-years (DALYs) [3].

Except for the increased risk of mortality and morbidity of stroke, much of the stroke burden arises from aggravated post-stroke disability [4]. Fifty percent of persons after stroke have residual deficits to some extent, such as physical impairments, cognitive impairments and generated psychosocial problems [5]. These residual deficits not only affect the quality of life of persons after stroke but also bring a great burden to the family system [6]. In China, approximately 80% of the persons after stroke survivors return home after discharge from the hospital with the need for support from family members. The traditional value of familism plays a vital role in the Chinese context and encourages and/or urges family members to care for their old and ill relatives. Additionally, from a global perspective, persons in need of help and support prefer living in their own housing instead of living in a nursing home or other care facilities [7, 8]. However, the sudden onset and need for long-term care means that family members do not have the ability to fully prepare for the new situation [9–11]. Lack of knowledge and practical skills in family caregivers, as well as inadequate support from healthcare professionals, have been found to negatively affect post-stroke recovery [12, 13]. Furthermore, long-term care requires that family caregivers dedicate a significant amount of time and energy, which is likely to negatively affect their physical and mental health, as well as well-being [9, 14]. Of note in previous studies, positive experience and effects of being a family caregiver were indicated by some family members, when some requirement has been achieved [23, 43]. Thus, it is significant to develop appropriate interventions and evidence-based policies to reduce the caregiving burden and improve the quality of life for both the persons suffering from stroke and family caregivers [15].

Prior to tailored interventions, an accurate assessment and understanding of the specific areas and magnitude of caregiver needs are essential [16]. In most cases, needs refer to the capacity to benefit from health care provision [17]. However, in some cases, there might be poor congruence between the needs of the individual and clinician judgements [18]. To identify the needs of an individual and receive insight about the magnitude of a specific need and whether the individual requires support or advice regarding that need, a need assessment based on the individual’s perspective should be performed. A comprehensive exploration of family caregivers’ needs is helpful to allocate social resources appropriately and prevent and reduce caregivers’ burden. It is of great importance for multidisciplinary professionals, social services, and policy makers to be able to provide such tailored services when necessary [19]. Globally, professionals and researchers have dedicated great attention to the needs of family caregivers of persons after stroke. However, such needs are multifaceted and experienced in different ways. For instance, studies from the U.S. and Australia indicated that obtaining information and being prepared for life after discharge were the most common concerns for family caregivers of persons after stroke [20, 21], while a study in Canada showed that needs interfacing with the healthcare team were the needs most reported [19]. In China, under the influence of traditional culture, the limited development of the primary healthcare system and the social care system of older persons [22] might result in other priorities of needs [9, 23]. However, research on the needs of family caregivers of persons after stroke in China is limited [24].

Hence, closely examining the self-perceived needs of family caregivers of persons after stroke in the context of Chinese culture is crucial, but so far it has not been sufficiently explored [9, 23, 25, 26]. Furthermore, most of the need-assessment tools used in research or in clinical settings in China are developed through cross-cultural validation, which means that the needs of family caregivers in these studies are predefined [27]. However, it has been highlighted that the incorporation of a mixed-method approach could further enhance the assessment quality [28]. Among the instruments developed for family caregivers of persons after stroke, the Caregiver Task Inventory (CTI) is a comprehensive inventory originally developed through mixed methods in the U.S. [29]. A modified and translated version (CTI-25) has been developed and used in Hong Kong [30]. However, it is unclear whether this instrument is suitable for family caregivers in mainland China, and further investigations are needed. Therefore, this study aimed to explore the needs of family members caring for stroke survivors in China using the CTI-25 as a framework for the analysis.

## Material and methods

### Study design

A qualitative research design was used to explore the needs of family caregivers caring for persons after stroke in China. The interview data for this study, analysed according to a deductive qualitative content analysis using the CTI-25 as a framework [31], have previously been used in a study aimed at inductively exploring the experiences of family caregivers caring for persons after stroke in China [23].

### Participants

Persons after stroke in three communities in Tianjin, China, were identified via community health management records by community nurses, between October 2014 and April 2015. A purposive sampling was used in this study where family caregivers were considered potential participants if they fulfilled the inclusion criteria: were 18 years or older, able to speak and understand Mandarin, had the main responsibility of caring for the person after stroke, was related by blood or marriage to the person and provided care free of charge. The characteristics of the 26 eligible family caregivers and the persons they cared for are presented in Table 1.

**Table 1:** Characteristics of family caregivers and stroke survivors

Family caregiver characteristics (*N* = 26)	
**Age, mean (range)**	63 (27–82)
**Gender**	
Male	6
Female	20
**Educational level**^**a**^	
Low	14
Medium	8
High	4
**Occupation**^**b**^	
Blue collar	16
White collar	9
Unemployed	1
**Length of care** in months**, mean (range)**	78 M (3 M-420 M)
**Relationship to stroke survivor**	
Spouse	21
Children	5
Daughter in law	1
**Stroke survivor characteristics (*N*** **= 26)**	
**Age, mean (range)**	71 (52–84)
**Gender**	
Male	19
Female	7
**ADL**	
Basically independent (Barthel ≥ 60)	5
Moderate dysfunction, needs help (41 ≤ Barthel ≤ 59)	7
Severe dysfunction, obviously dependent (21 ≤ Barthel ≤ 40)	5
Completely dependent (Barthel ≤ 20)	9
**Present during the interview**	
Yes	11
No	15

^a^ Low: elementary school or low vocational education. Medium: secondary school or intermediate vocational education. High: higher vocational education or university education

^b^ Blue collar: manual worker; white collar: clerical worker

### Data collection and procedures

All potential caregivers were first contacted by the community nurses and informed about this study. Once verbal consent was obtained, the first author acquired the caregivers’ phone numbers from the community nurses and made appointments for the interviews. All interviews were performed by the first author, whom is a nurse with experience in stroke care. Participants had the right to choose where to be interviewed and to withdraw from participating at any time. Written informed consent was obtained from each of the participants before the interview. All invited family caregivers (*n* = 26) accepted participation, and no one withdrew during the study process. The interviews were recorded by a digital audio recorder and field notes were written both during and directly after each interview. An interview guide was used, and all interviews started with the broad question “What is your experience of caring for your relative who has experienced a stroke?” When describing their experiences, participants usually also expressed needs and what kind of support they wanted. However, to receive deeper knowledge regarding needs, a follow-up question was asked: “What are your needs during caring for your relative who has experienced a stroke?” To encourage the participants to generate more information and maintain rich data, questions such as “What happened next?”, “What were you thinking then?”, “How did that effect you?”, and “What do you mean by that?” were also asked during the interviews. To test the interview guide, three pilot interviews were performed, which only resulted in minor adjustments. Therefore, these interviews were included in the analysis. The median length of the face-to-face interviews was approximately 50 min (range 28–136 min). Ethical approval was obtained from the Tianjin Medical University Ethical Committee (TMUEC201400202), China.

### Data analysis

A deductive qualitative content analysis [23] was used to reveal if the needs expressed by the interviewed family caregivers were in line with the assessment scale: CTI-25, the Chinese version of the modified Caregiver Task Inventory. The CTI-25 consists of 25 items divided into five subscales focusing on *learning to cope with new role*, *providing care according to care-receiver’s needs, managing own emotional needs, appraising supportive resources, and balancing caregiving needs and one´s own needs* [32]*.* According to the subscales and included items of the CTI-25, a categorization matrix was created and used in the analysis. The categorization matrix also included a section called *Other needs* where identified needs not belonging to the CTI-25 could be included. Both identified met and unmet needs were analysed. Moreover, the number of meaning units for each item was counted and presented.

The analysis began with all 26 recorded interviews being transcribed verbatim by the first author. Another author checked the transcripts while listening to the recording to ensure their accuracy. To make the data available to all authors, three interviews were translated into English by the first author, who is fluent in both English and Mandarin. Furthermore, the first author identified meaning units in these interviews. These interviews, together with the identified meaning units, were then discussed by all authors to ensure that the analysis focused on the needs of family caregivers rather than just experiences, feelings, or relations. The first author then continued with identifying meaning units regarding needs in the remaining 23 interviews and translated the meaning units into English. Three of the authors then read and arranged all meaning units according to the developed categorization matrix based on the CTI-25. All the meaning units were allocated to different subscales and into the respective items (Table 2). All identified *other needs,* 23 meaning units, focused on financial needs, and therefore, these needs were labelled and presented as *direct and indirect financial needs.*

**Table 2:** Categorization matrix including number of meaning units for each item in CTI-25

Sub-scale	Item	Number of meaning units	
Learning to cope with new role	Monitor course of condition and evaluate significance of changes		7
	Normalize care-receiver routine, within bounds of the impairment		5
	Perform basic ADL for the care-receiver		3
	Gain knowledge about the disease		50
	Cope with the loss/restriction of future family plans		8
Providing care according to care-receiver’s needs	Being available when needed		3
	Supervise prescribed treatments and general recommendations		4
	Evaluate strength/resources of the care-receiver		5
	Cope with upsetting behaviour of the care-receiver		9
	Give appropriate consideration to care-receiver’s options and preferences		6
Managing own emotional needs	Resolve guilt over ‘negative feelings’ towards care-receiver		8
	Find a locus of blame for the condition/disease		3
	Separate feelings regarding condition from feelings towards the care-receiver		5
	Resolve uncertainty about one’s skills as a caregiver		5
	Release tensions/feelings towards the care-receiver		28
Appraising supportive resources	Anticipate needs for future assistance		77
	Designate other responsible caregiver(s)		18
	Manage feelings towards other family members who do not regularly help		12
	Maintain the family as effective decision-making group over a long period of time		3
	Interact with medical, health and social service professionals		28
Balancing caregiving needs and own needs	Satisfy needs for creativity/originality to offset tedious routines		17
	Avoid severe drain on physical strength/health		29
	Make up for or avoid loss/restrictions on future plans and perspectives		8
	Readjust personal routines		26
	Compensate for disruption of sleep		15

## Results

The five subscales and the respective items in the CTI-25 — describing the needs of family caregivers — were identified. Family caregivers also expressed direct and indirect financial needs to a high extent. Subscales, items, and the number of meaning units are presented in Table 2.

### Learning to cope with a new role

The family caregivers described the need for learning to cope with their new role since they encountered new situations with unfamiliar tasks and challenges when taking care of their relative after a stroke. The strongest need of family caregivers was to gain knowledge about the disease, including treatment such as medicine and rehabilitation, as well as about the possible complications that could occur. Some family caregivers stated that they completely lacked knowledge, even long after the stroke.

*“For the entire time, we didn´t know how he could have this disease. We didn´t know what a stroke was even after he had a stroke.”* (N16, Wife).

Family caregivers expressed needs regarding monitoring and evaluating changes in the health status of their relatives, which aimed to identify if the health or life of the persons after stroke were at risk, but also served as proof that the caring and support provided by the family caregivers was correct. However, the participants thought that the professionals or other specialist agencies did not pay enough attention to this need or provide effective support for them, which led to stress, anxiety and insecurity about the care they provided.

*“I feel there must be something wrong with him. I always observe him. Is he having dementia? Or any kind of stroke complication? Depressed? Am I right? Otherwise, why is he always restless like a child with ADHA (attention deficit hyperactivity disorder)? I care for him every day with my own judgement. I am not sure whether it is right or not, I just go with my instinct.”* (N2, Wife).

The new situation required family caregivers to take on more responsibility than before. Some family caregivers described that supporting relatives performing basic activities of daily life was very much in focus, especially regarding going to the toilet and cleansing. However, family caregivers strive to continue living their regular life and to do so, the recovery of the person is crucial. One family caregiver explained that:

*“My needs? My needs were that he could be recovered. If not, at least that he can take care of himself. Eat, drink, go to toilet, just live regularly and normally. [Then] everything will be all right for me.”* (N24, Daughter).

Consequently, there was a need to normalize the persons’ routine within the bounds of their impairment, since that resulted in life becoming more relaxed and easier for the family caregivers.

### Providing care according to care-receiver’s needs

The family caregivers faced difficulties providing care according to the needs of the person with stroke. One example was the timing problem, which meant that it was hard to provide care immediately when asked for. Another example was the challenge of supervising recommended treatments and care because of limited knowledge and professional support. Moreover, the family caregivers had to balance doctors’ advice and the preferences and decisions made by their relatives with stroke:

*“…I asked him “Do you sleep well at night?” His answer was no, then I asked him to take the sleeping pills. He then changed his answer to yes and refused to take those pills, not even one tablet. He just hates to take pills. What can I do? This medicine was prescribed by the doctor, and it is good for improving brain functions. I tried my best to talk to him about the pills, but he still refused to take it. Those pills are wasted. He insisted that he has been doing well without it.”* (N22, Daughter).

Upsetting behaviour, such as aggression, also affected the family caregiver’s ability to perform care and was considered one of the main problems to manage. Family caregivers stated that the most upsetting behaviour was when the relatives with stroke did not cooperate with the rehabilitation and lacked the motivation to perform rehabilitation exercises. For instance, one woman described that she is doing the best she can to try to help her husband:

*“I know nothing about rehabilitation, I just think that walking exercise is the way for his recovery, but he doesn’t want to do it. Whenever he gets home, he always takes off his socks and spends his time lying in bed. I repeatedly asked him to do the walking exercise, then he got very upset and angry. He just doesn’t want to do any exercise. I told him this is what I can do. I tried my best for him, that's just the way it is.”* (N20, Wife).

The family caregivers had difficulties identifying how much help and support the person with stroke needed, which resulted in the family caregiver experiencing a burden. Moreover, the kind of welfare or resources that were available for persons after stroke, as well as the possibility of applying for support, were unclear to the family caregivers. This made it difficult for them to arrange their lives and had a significant negative effect on them both physically and psychologically.

*“I mean, life gets better when he gets better. I don’t know how much he can do in taking care of himself eventually. The more he can do for himself, the easier my life will be. It is not just about what matters to me, he´s worried about himself too. If he can do things by himself, it will save me some work.* (N10, Wife).

### Managing one’s own emotional needs

Due to taking care of their relatives with stroke, the family caregivers felt tension almost all the time and expressed feelings of emotional stress and anxiety. Most of the time, however, the family caregivers did not know how to deal with their negative emotions or how to ask for help. They often lacked having someone to listen to them, but they also said that they did not know how to describe their tensions and feelings to others.

*“I´m so stressful every day, there is no one else can help at home. We only have one child, and he´s not available all the time. Oh, well! Words can’t describe how I feel, I can’t help but just want to cry…”* (N16, Wife).

Family caregivers indicated that they sometimes had negative feelings due to taking care of their relatives who had suffered from stroke. Meanwhile, the family caregivers also tried to distinguish between feelings about the disease and the condition from their feelings towards the person with stroke, but found it difficult.

*“My son is 30 years old; he´s still single because we don’t have money to buy an apartment. Now, his father is handicapped. I even grumble at my husband sometimes. Other parents can work and buy the apartment for their sons, but he can’t. Well! He doesn’t want to be like this either. I just can’t stop grumbling at him.”* (N23, Wife).

Confusion, anxiety, and anger about the current situation resulted in family caregivers wanting to find someone to blame for what had happened, including the person with stroke. Sometimes they reached the point that they thought the only thing that could end this horrible life was the death of their partners. Such feelings and thoughts caused guilt and made the family caregiver feel remorseful.

*“…sometimes, I can´t control myself well. ‘Just die’ I shout at him. ‘Go and die, die, to die…Why don’t you die? Do you want to live? If you want to, just do well.’ After I shouted, I looked at him, and thought how pitiful he was. I still have to take care of him. It is my duty…”* (P23, wife).

Some family caregivers even doubted their skills as caregivers. They reflected and looked for some mistakes during caring. They were not sure that the care they gave to their relatives was good or correct, especially when the condition of their relatives worsened.

*“Sometimes I blame with myself. Do I take good care of him? No, I don´t. If I’ve done everything right, took good care of him, he wouldn´t be in such a mess, right?”* (N15, Wife).

### Appraising supportive resources

The situation, including the family caregivers’ negative emotional experiences, resulted in them seeking support, both from other family members and friends as well as from society. For instance, one participant explained:

*“If there is an institution that can help me to take care of my mom during the day, then I could go to work. In China, we don’t have a place to do that. Being honest, I think we should invest more in community development. Now ordinary people like me have to depend on ourselves to solve the problem. If we could find someone or organizations to provide supportive services, that would be great. Unfortunately, we don’t have such resources.”* (N1, Son).

Worries and unsafe feelings about the future were commonly expressed by the family caregivers, who explained that they were getting older and would start to need care themselves. This would eventually result in their being unable to take care of their relatives. Family caregivers, therefore, expected that they would need more assistance from others in the future, both from other family members and from formal health and social care.

As a result of the relative having a stroke, the original and stable family decision-making system has been destroyed. This means that the family caregivers had to make more decisions independently, especially medical decisions. Therefore, the need for support from professionals, such as staff working in the medical, health and social service sector, became more important for family caregivers, which was especially the case for female caregivers:

*“I really need a doctor who can tell me what has happened to my husband…I hope that a doctor will be available whenever I need help. He can answer my question and tell me what to do. I really have no idea what to do and who to ask for help, especially in emergency situations. He is very fragile; I am afraid to call 120 (Emergency number in China) sometimes. So, I hope someone can help me with those medical questions and tell me what to do in case of emergency. Besides, it would be great if the community hospital could offer home infusion services”.* (N3, Wife).

Most family caregivers were willing to accept responsibility for caring for their relatives with stroke. However, some of them paid close attention to the efforts and contributions made by other family members to the caring, especially close relatives. This attention often left them feeling unsatisfied and resulted in mood swings. There was a need to find ways to deal with the negative feelings towards other family members who did not regularly help, as described by family caregivers during interviews, including this wife taking care of her husband:

*“My kids are not helpful; they all count on me in taking care of their dad. If he can do better in recovery, then my work would be easier. In fact, I have to bear the burden only by myself. We have three kids; it is not a small family for now or even in the past. But, none of the kids share the burden with me. Well, how can I not be angry?”* (N14, Wife).

### Balancing caregiving needs and own needs

To be able to care for the relatives suffering from stroke, the family caregivers had to readjust their personal routines and activities based on their relatives’ situation. Family caregivers described efforts they made to accept the loss and restriction on future plans and perspectives as well as the loss of self-actualization and fulfilment.

*“I like exercise. I have practised martial art since 1980. I thought about changing unhealthy habits and doing more exercises after retirement. You know, I mean exercises like sit-up, parallel bar, and dumbbell. Good health depends on exercise. However, I´m stuck in taking care of her now, I couldn’t find time to do exercises. What else can I do to practice?”* (N25, Husband).

The severe drain on physical health and disruption of sleep were frequent problems for family caregivers. Many family caregivers described that the hurt and pain were caused by taking care of personal hygiene for the person with stroke. Night sleep was disturbed and affected by caring and worries. The family caregivers wanted to be free from the terrible routines of care. Even though the family caregivers were aware of these problems, they lacked a way to cope with them.

*“I wish I could have a little free time, but that´s impossible. I have devoted myself entirely to her. I have to get back soon even when I go for grocery shopping. I have no personal life at all even though I wish I could have it. She complains to me whenever I go out for things like shopping food.*” (N5, Daughter).

### Direct and indirect financial needs

To a high degree, family caregivers described the needs related to financial support. Almost all family caregivers experienced a financial burden caused by their relatives’ stroke, which involved both direct and indirect financial needs. Medical insurance was described as support for large and long-term costs of treatment and medicine — the main cause of direct financial costs. However, the family caregivers had a strong belief that their relatives with stroke would recover. Hence, they were willing to buy health-care products and treatments, such as massage, which were not covered by medical insurance. Moreover, the stress of indirect financial needs was also emphasized by family caregivers; the income of the whole family had fallen significantly, not only because the relative with stroke could not work but also because at least one family member dedicated day and night to caring tasks and therefore was also unable to work. This was explained in the following way by one family caregiver:

*“My son shared some expense for his father’s hospital bill. Otherwise, it´s hard to pay the bill with the salary from two of us. We do have medical insurance, but there are many expenses that are excluded from the coverage. It´s a burden on us. Well! We do everything for his father’s recovery. I already cut my budget on dressing and social activities. For my age, it doesn’t bother me any more about dressing. So, I try to save money as much as I can. My daughter in law always says that I´m too stingy. But you know, I must tighten the budget because our salary is not enough to cover my husband’s medical expense. To be honest, our pensions are enough to cover daily living costs. But now he has been sick, and we have to pay the medical expense regularly. It costs a large portion of our incomes every month.* (N20, Wife).

## Discussion

This study explored the needs of family members caring for stroke survivors in China using the CTI-25 as a framework for analysis. As a result, coping with the new role, providing care according to care-receiver’s needs, managing their own emotional needs, appraising supportive resources and balancing caregiving needs and one’s own needs areas were all expressed by the participants. Moreover, the analysis revealed that family caregivers expressed other needs not part of the CTI-25 that were related to direct and indirect financial needs.

“Anticipate needs for future assistance” was the most common expressed need in the present study. The interviewed family caregivers were mainly middle-aged and older, with an average age of 63 and a 78-month average length of care. As family caregivers age and care time is extended, there is an increased risk of health and functioning decline [34]. Additionally, caregiving seems more complex, and the burdens of care may be greater by comorbidities and other impairments compared to younger caregivers [35]. They are thus increasingly dependent on assistance from the community or other family members and friends. Furthermore, being at increased risk of disability and death, they themselves have awareness of the finitude of life [36]. Therefore, appraising supportive resources and anticipating needs for future assistance is truly one of the most important needs to meet.

For needs on knowledge about the disease, similar results have been reported before, though with different wording, such as educational needs, training needs, knowledge regarding preventing recurrent stroke, and understanding the effects of medications [27]. It is widely acknowledged that having a good command of disease knowledge, having more access to informational support and greater knowledge about stroke care can contribute to more effective problem-solving skills and quicker adaptation to the caregiving role [37]. However, family caregivers of stroke survivors often receive inadequate support from health professionals and frequently feel abandoned and unrecognized by the health care system. In China, this might, for instance, be related to both the organization of health care and the dearth of primary health professionals [39, 40]. The workloads and disproportionate number of health professionals may leave family caregivers in a neglected position. Consequently, it is of importance to develop new ways to support family caregivers. For instance, as pointed out in a recent review internet-based support interventions may be an option to close the support gap for informal caregivers [41]; they can not only reduce the cost but also have a role in reducing the social isolation that can come with caring [41]. In the light of COVID-19 it seems as internet-based interventions have promising potential in providing support for family caregivers at home in China.

How to deal with negative emotions is another need expressed by family caregivers. They sometimes generate emotions such as anxiety, guilt, or anger during the caregiving process. While most of the time, they have no one to pour their hearts out, they don’t know how to release this tension or even how to describe their feelings. In the Chinese cultural context, filial piety and marital obligations encourage family members to be the main caregivers and to try their best to care for their relatives with stroke [9]. This feeling of responsibility has also been acknowledged in other cultures [43–45]. Kitzmüller et al. [46] found that the rooted culture may force family caregivers into caregiving, resulting in them taking on a task they do not truly want. The conflict family caregivers might experience when trying to do their best with this undesirable task can cause both negative emotional and physical symptoms such as guilt, blame, uncertainty, sleeping difficulties and increased risk of chronic diseases [47, 48]. Psychosocial and existential support is the predominant approach to family caregiver intervention research [49, 50]. Additionally, the combination of assistive technology, such as alarms, smartphones and telehealth system, to support caregivers in improving caregiver distress, reducing caregivers’ burden and cutting down on health care costs proved to be highly cost-effective [50].

Financial needs were emphasized by family caregivers in the present study and are the only need that is not a part of CTI-25. As the results showed, financial burden was common and related to stroke, which involved direct costs (e.g., expense on treatment and medicine) and indirect costs (e.g., the possibly dropped income resulted in all-day caring tasks). Additionally, other researchers have identified medical expenses as a main stressor for families of persons after stroke in mainland China [51, 52]. For instance, Liu, Zhou, and Wu [53] reported that, compared to the average personal monthly income of 3,357 RMB, the monthly rehabilitation expenses for persons after stroke were more than 4,000 RMB. Many family caregivers have described that employment becomes difficult and even impossible when also caring for a person with stroke, resulting in an even greater financial burden. Such financial burden could be a source of other caregiving burdens and needs of family caregivers [54]. In recent years, the Chinese government has been devoted to providing equal access to basic health care with reasonable quality and financial risk protection in response to potentially catastrophic health expenditures and impoverishment [40]. Notably, the government quadrupled its funding for health from 2009 to 2017. Currently, a long-term health sector strategy, Healthy China 2030, has been launched. Despite this, the assessment of financial needs should not be ignored when developing assessment tools.

Of note, direct and indirect financial needs are not part of the CTI-25. However, this is included in the original version of the CTI, which was developed in the U.S. and consists of 45 items [29]. In the original version, assumed financial costs (actual and potential) were identified as an item related to financial needs. When modifying and contextualizing the instrument for family caregivers’ needs in Hong Kong, these items were excluded based on an evaluation from an expert panel. This might be related to selection bias and the fact that only one family caregiver was involved in the expert panel during modification [30], while the present study included the voices of 26 family caregivers. During the development or the cross-cultural validation process of an instrument, some originally designed items could be excluded from the methodology and cultural considerations [54]. For instance, financial items might have been excluded since questions about financial status are included in another instrument used together in the same survey [55]. Furthermore, in many cases, participants might hesitate to describe privacy issues such as economic level [56] and therefore give ambiguous or inaccurate answers in questionnaires, and items might therefore be excluded. However, in a qualitative study such as this one, the interest shown in the persons’ experiences and needs could have resulted in an increased willingness to discuss this aspect to a greater extent.

To summarize, stroke is sudden and often an unexpected event affecting the whole family [9], and family caregivers often have to undertake the demanding caregiving role suddenly without forewarning, resulting in them facing new and challenging life situations. The ecological resilience framework, developed by Windle and Bennett, showed that factors from the individual, community and society levels are all the cause of the various needs [57], which could also be regarded as the shortcut to satisfy their needs. In this study, factors such as own health problems and employment status at the individual level, health resource accessibility at the community level, and medical expenses at the social level seems to be important factors that cause needs to be generated. Additionally, family caregivers are often accompanied by different types and levels of needs. Comprehensive assessment of needs is therefore crucial to develop the most cost-effective interventions to improve care, consequently ensuring that patients and their families experience minimal unmet needs and improve or maintain their health and wellbeing [58, 59]. In addition to the present needs of family members caring for stroke patients, this study might also contribute to the refinement of an assessment tool that is in line with the reality of family caregivers. Additionally, whether to add financial needs in an instrument suitable for family caregivers in mainland China needs to be tested. Regardless of how the assessment tool looks, it is always significant to also discuss this with family caregivers so they become involved in the care and can describe their needs with their own words in order to design a tailored intervention.

### Limitations

There are some limitations to this study that could affect its trustworthiness and therefore should be highlighted. Firstly, out of ethical and safety considerations, the person with stroke was present during some of the interviews (*n* = 11). The reason for this was that family caregivers did not think it was appropriate to leave their relatives unsupervised. This resulted in the persons after stroke sometimes answering questions during the interviews. These data, however, were excluded from the data analysis since it was not directly the perspective of the family caregivers. There is a risk that the presence of the person with stroke could have prevented the family caregiver from speaking freely. However, a little bit unexpectedly, negative emotions and caregiving needs were also described in these interviews, which overall, were similar to the interviews in which only the family caregiver was present. Therefore, all the interviews were included in the study.

The need assessment tool (CTI-25) was used as a framework in the analysis using a predefined categorization matrix means there might be content in the interviews that does not fit into the framework. To ensure trustworthiness of the results [33], we therefore also analysed identified needs that were not part of CTI-25. This helped to highlight important content related to the needs in a mainland Chinese context that otherwise would have gone missing. All items in the CTI-25 were represented in the analysis, but financial needs were also expressed in the interviews which is not part of the CTI-25. Moreover, it has been reported that there is always more than one possible interpretation of a text, which is also the challenge of content analysis — there is no standard or formula or ‘right’ way of doing it [31] which gives the author(s) an import role in the analysis. Sometimes it was difficult to decide which item some of the meaning units belonged to, that is, a meaning unit may contain views related to several items. Hence, several meetings were held among the members of the research group to discuss and check interpretations. The topic of the first meeting was to ensure that the analysis focused on the needs of family caregivers rather than experiences, feelings, or relations. Furthermore, as much information as possible, such as characteristics of participants and quotations of the interview, was provided in the context to make it possible for readers to assess the transferability into other contexts and settings.

## Conclusion

This study identified that family caregivers of stroke survivors in China had various needs, which is important to acknowledge when assessing needs and improving health care for family caregivers. The family caregivers expressed needs in relation to all subscales and items in the CTI-25 but also stated financial needs to be of great importance. Cultural adjustments and modifications should be made if the CTI-25 is used in mainland China. This study also indicated a comprehensive and holistic perspective (individual, community, and social level) when identifying, assessing needs or implementing interventions to support family caregivers.

## Data Availability

The datasets used and/or analyzed during the current study are available from the corresponding author on reasonable request.

## Abbreviations

CTI 25: Caregiver Task Inventory-25
